# Anatomical variability of the lateral pterygoid plate and its influence on the dimensions of infratemporal fossa in human skulls

**DOI:** 10.1038/s41598-025-26776-6

**Published:** 2025-11-28

**Authors:** Pavan Sohal, Aisha Zeeshan, Femina Sam

**Affiliations:** 1 Human Anatomy Unit, Department of Biomedical Sciences, School of Infection, Inflammation and Immunology, College of Medicine and Health, University of Birmingham, Birmingham, B15 2TT UK; 2 School of Medicine, Medical School, University of Nottingham, Nottingham, NG7 2UH UK; 3 Department of Anatomy, Christian Medical College, Affiliated to The Tamil Nadu Dr. M.G.R. Medical University, Chennai, Vellore, 632 002 India

**Keywords:** Lateral pterygoid plate, Articular tubercle, Infratemporal fossa, Foramen ovale, Medial pterygoid plate, Trigeminal neuralgia, Anatomy, Diseases, Medical research

## Abstract

The lateral pterygoid plate is a key landmark within the infratemporal fossa, and its morphological variations influence surgical access to the foramen ovale. This study evaluated the morphometry of lateral pterygoid plate and its spatial relationships in 60 dry skulls. The specimens of skull were categorized into direct, lateral, removed, and medial types. Measurements included five sectional breadths, height, distances from lateral to the medial pterygoid plate, the anterior tubercle, and degree of lateral deflection. The specimens of skull classified as medial type had significant greater mid-to-proximal breadth and height of lateral pterygoid plate. Increased distal breadth correlated with reduced lateral pterygoid plate-articular tubercle distance in the specimens of skull classified as removed and lateral types, with a moderate and strong inverse correlation in both groups. These specimens of skull also showed a narrower infratemporal space due to lateral positioning of the lateral pterygoid plate, despite no significant increase in breadth or height. The specimens of skull classified as removed type remained to be obstructive due to the added shortcoming of a deficient posterior border of lateral pterygoid plate. These findings highlight that the lateral pterygoid plate significantly affects the dimensions of the infratemporal fossa. Since the lateral and removed types of the lateral pterygoid plate are found in nearly half of the individuals, clinicians should be aware of these variations when accessing the foramen ovale during trigeminal neuralgia interventions.

**Supplementary Information:**

The online version contains supplementary material available at 10.1038/s41598-025-26776-6.

## Introduction

 The infratemporal fossa (ITF), located on the inferior aspect of the cranial base, is a region of clinical relevance across various surgical disciplines. It houses key foramina namely the foramen ovale (FO), foramen spinosum (FS), and the foramen of Vesalius (FV) which transmit vital neurovascular structures and are approached with caution during surgery due to the risk of intraoperative complications^1–3^. Projecting downward from the junction of the sphenoid body and greater wing are the pterygoid processes, consisting of the medial and lateral pterygoid plates, separated posteriorly by the pterygoid fossa. The lateral pterygoid plate (LPP) a broad, thin, and everted bony structure tending posterolaterally, serves as an attachment for the pterygoid muscles and contributes to the skeletal framework of the ITF^2–6^. Importantly, its posterior border lies in close alignment with the FO, a feature of major surgical relevance. Although the ITF has been accessed for a variety of surgical interventions, one notable approach targets the trigeminal ganglion through the FO for the treatment of trigeminal neuralgia^2,7^. Previous studies have focused primarily on the internal morphology and neurovascular relations of the FO^8–10^, whereas external adjacent bony structures such as the LPP that potentially influence cannulation success remain underexplored.

Elnashar et al. (2020) were among the first to suggest that the LPP can obstruct the medial portion of the FO, complicating cannulation^9^. Given its proximity to the FO and invisibility under fluoroscopy, the LPP warrants consideration during percutaneous and neurosurgical approaches to the ITF^11^. These underscore the importance of understanding the spatial relationship between the LPP and FO during neurosurgical and maxillofacial procedures. In 2020, Iwanaga et al. proposed a classification system based on the spatial relationship between the FO and LPP: direct, medial, lateral, and removed. In the specimens of skull classified as removed type, the posterior border of the LPP is insufficient to guide needle trajectory, often necessitating redirection^4^. This suggests that variations in the morphometry of the LPP can alter the surgical corridor and reduce the space within the ITF, thereby complicating access and increasing procedural challenges.

Therefore, this study aimed to perform a detailed morphometric analysis of the LPP and evaluate its relationship with adjacent anatomical landmarks across the specimens of all four types of skulls. It was hypothesized that the specimens of skull classified as the “removed” type would present a higher risk of cannulation difficulty or obstruction to FO access. By providing quantitative data and anatomical correlations, this study sought to enhance preoperative planning and optimize needle trajectory during percutaneous procedures involving the FO.

## Materials and methods

### Collection of samples

**Fig. 1:**
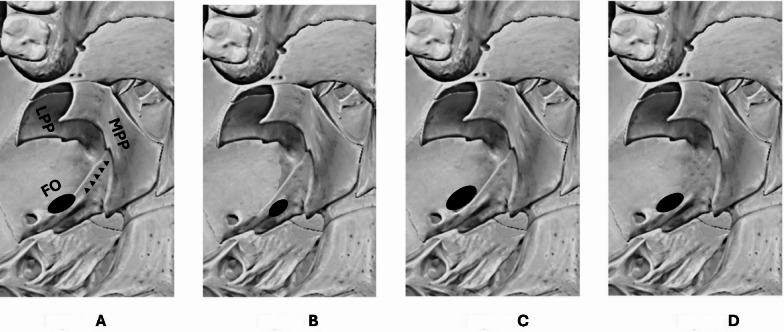
Classification of the specimens of skulls based on the spatial relationship of the LPP and the FO. Figure A to D depicts the schematic picture of the specimens of skull classified as direct type, lateral type, medial type and removed type respectively. LPP – lateral pterygoid plate, MPP – medial pterygoid plate, FO – foramen ovale, black arrow heads pointing the posterior border of the LPP.

A total of 38 dry adult human skulls (76 sides) of unknown age, sex and ethnicity was procured from the bone collection of the Human Anatomy Unit, University of Birmingham. Sixteen sides (nine right sides and seven left sides) were excluded from the study due to signs of bony attrition or damage caused due to manipulation on the lateral and medial pterygoid plates, and a remaining total of 60 sides were utilised for this study. Each side (left and right) of the specimens of skull was classified into one of the four skull types: type I, type II, type III and type IV based on the spatial orientation of the FO to the LPP. The classification system used in this study was slightly modified from the original description provided by Iwanaga J et al.^4^ (Table 1; Fig. 1).

**Table 1:** Classification guidelines for the specimens of the different types of skulls. The table outlines the description outlined by Iwanaga et al. based on the LPP–FO spatial relationship and the anatomical description of the classification system followed in the present study with the number of specimens of skull utilized in each type.

Classifications of the specimens of skull	Lateral (Type I)		Medial (Type II)		Direct (Type III)		Removed (Type IV)	
**Anatomical description by Iwanaga et al.**	Posterior border of the base of the LPP ends at or close to the lateral border of the FO		Posterior border of the base of the LPP ends at or close to the medial border of the FO		Posterior border of the base of the LPP ends at or close to the centre of the FO		Posterior border of the base of the LPP ends distant from the FO	
**Anatomical description in the present study**	Posterior border of the base of the LPP lies lateral to the FO, regardless of the distance		Posterior border of the base of the LPP lies medial to the FO, regardless of the distance		Posterior border of the base of the LPP lies 0 to 8 mm close to the FO		Posterior border of the base of the LPP lies more than 8 mm from the FO	
**Number of sides of the specimens of skull in the present study**	Right	Left	Right	Left	Right	Left	Right	Left
	9	6	8	7	7	8	7	8

## Measured parameters

**Fig. 2:**
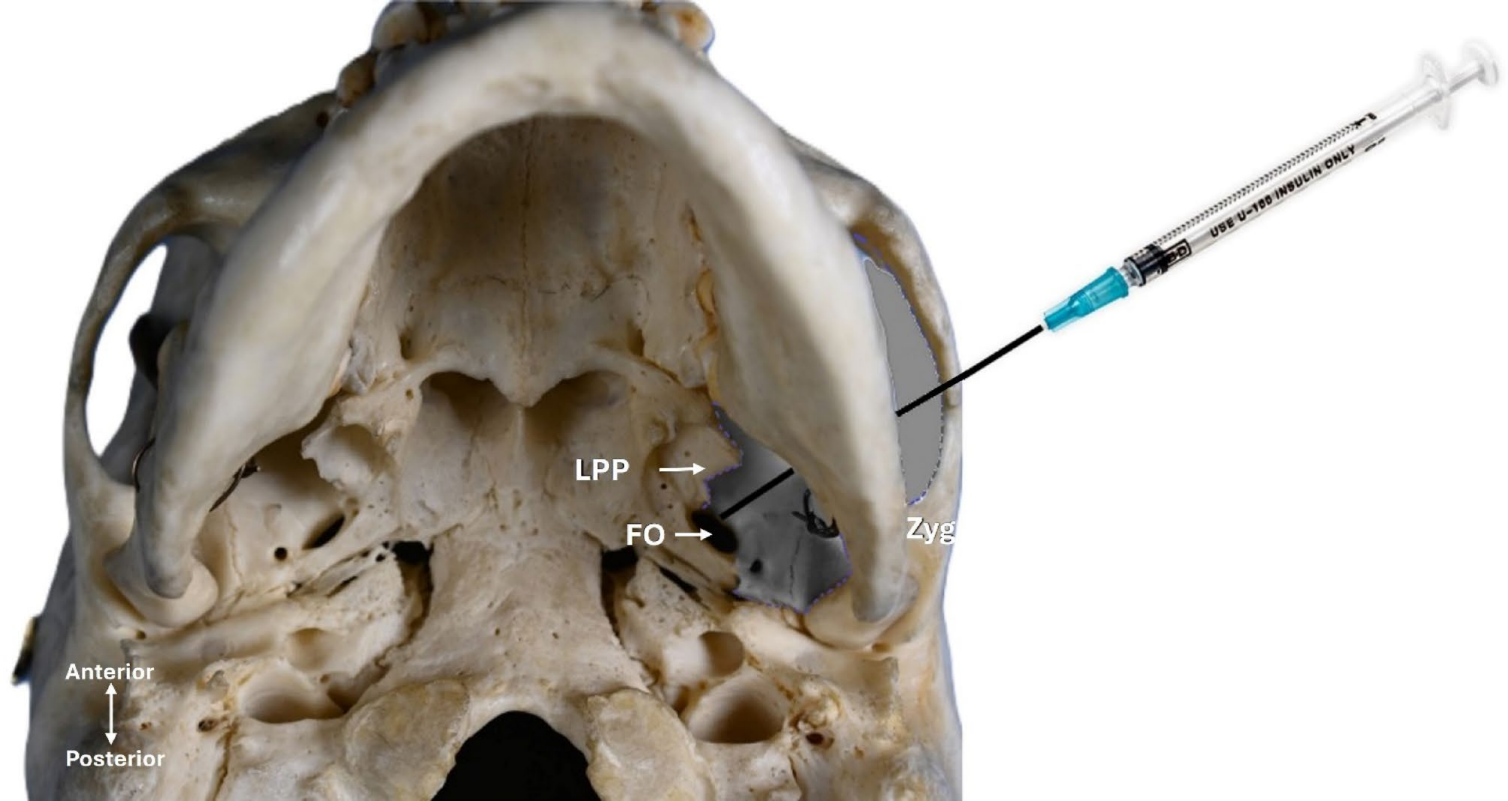
Infratemporal fossa corridor. The illustration on an inferior view of a specimen of skull demonstrating the orientation of a needle directed toward the FO, representing the pathway used for percutaneous approaches to the trigeminal ganglion. LPP-lateral pterygoid plate, FO-foramen ovale, Zyg-Zygomatic bone, the outlined and highlighted region represents the ITF corridor.

To illustrate the clinical relevance of the measurements, a conceptual depiction of the needle position during percutaneous intervention in trigeminal neuralgia is shown on a specimen of skull (Fig. 2).

**Fig. 3:**
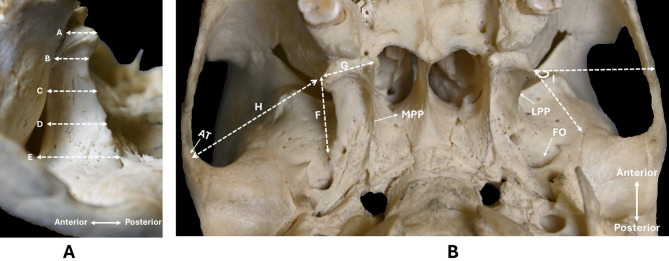
Images showing the parameters measured on the base of skull. Figure A shows the right-side of a skull showing the measurements at 5 sections of LPP (A – E). Figure B shows the parameters exploring spatial relationships of the LPP, (F - I). A-I denotes the parameters 1 to 9. Each white dashed line shows the distances measured and white arrows show anatomical features. FO-foramen ovale, AT-articular tubercle, MPP – medial pterygoid plate, LPP – lateral pterygoid plate.

Nine morphometric parameters were assessed on each side of skull to acquire data on the LPP and its spatial relationship with the neighbouring osseous anatomical structures within the infratemporal fossa as they are described in Table 2 and are illustrated in Fig. 3.

**Table 2:** Overview of morphometric parameters measured in this study and their anatomical descriptions. The table presents the precise anatomical landmarks used to measure each parameter in this study for easy reproducibility.

No	Parameters	Anatomical description of the measurement points
1	A- Breadth of LPP at the most distal region	From the most distal point of the posterior border of the LPP to the distal point of the pterygomaxillary fissure
2	B- Breadth of LPP at the midpoint between the distal and the middle region	Midpoint between parameters 1 and 3
3	C- Breadth of LPP at the middle region	From the midpoint of the posterior border of the LPP to the point where it joins the posterior margin of the pterygomaxillary fissure
4	D- Breadth of LPP at the midpoint between the proximal and the middle region	Midpoint between parameters 3 and 5
5	E- Breadth of LPP at the most proximal region	From the most proximal point of the posterior border of the LPP (closest to FO) to the posterior border of the pterygomaxillary fissure
6	F- Height of LPP	Proximal to distal points of the posterior border of the LPP measured longitudinally
7	G- Distance between LPP and MPP	Shortest linear distance between the distal points of LPP and MPP
8	H- Distance between LPP and AT	Linear distance between the apex (most distal point) of LPP and the AT
9	I- Degree of lateral deflection of LPP	Angle between a line along the distal margin of the LPP and a horizontal line extending from the distal end of the LPP to the zygomatic arch

## Methods of measurement

**Fig. 4:**
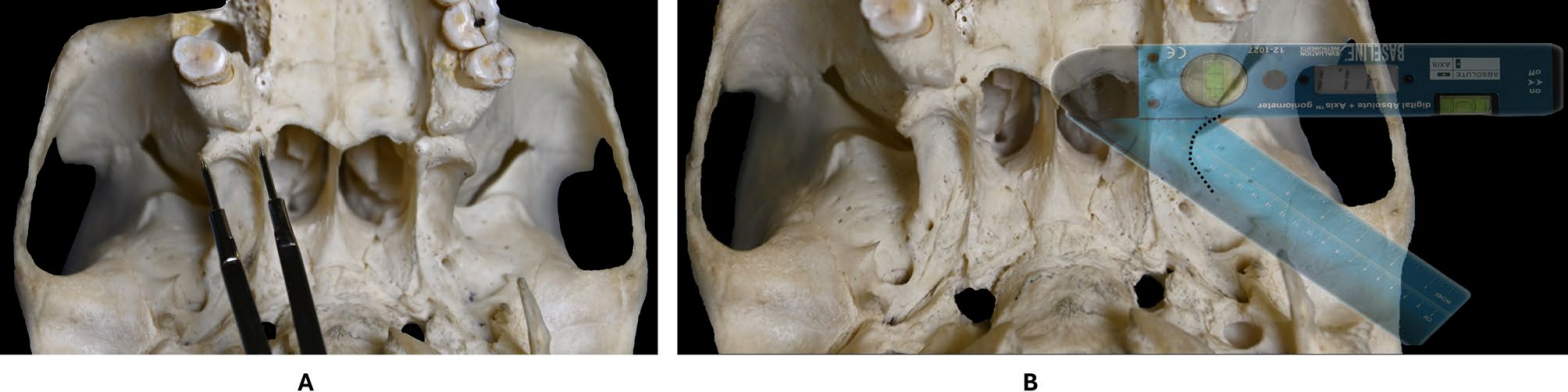
Inferior aspect of the base of skull showing the measurement techniques for parameters 7 and 9. Figure **A** shows the measurement of parameter 7 using a two-pin metal spacer. Figure **B** shows the specimen of skull with a digital goniometer to measure the degree of lateral deflection of LPP. The transparency of the digital goniometer has been reduced to improve the visualization of the underlying structures. The posterior border of the LPP is marked by black dotted line.

The parameters 1 to 8 were measured using an RS Pro electronic digital vernier calliper (Manufacturer’s Code: 49-923-255 C) with a maximum measuring range of 200 mm and an accuracy of ± 0.03 mm and the angular measurements for parameter 9 was obtained using a Baseline Digital Absolute + Axis Goniometer (Model Ref: 12-1027) with a working range of 0 − 185°, angular resolution of 0.1°, and an angle precision of ± 0.5°. Both the calliper and goniometer were calibrated to 0.00 mm and 0.0°, respectively, prior to each use to ensure measurement accuracy. In the specimens of skull where the confined anatomical space, particularly within the infratemporal fossa, hindered the direct calliper use, a metal two-pin spacer was used to span the target landmarks. The distance between the spacer tips was then measured using the digital calliper to obtain accurate readings (Fig. 4).

All the specimens of skull were classified into four types independently by two observers (FS, AZ), who were fully blinded to each other’s decisions. During this process, the authors did not encounter difficulties in classifying skull specimens as lateral or medial types, as this was evident from the position of the LPP relative to the FO irrespective of how much it was away from the FO. For the specimens of skull classified as direct and removed types, in borderline cases, where the measured distance was close to 8 mm, the classification was determined by consensus. Once the specimens of skull were categorised, all the measurements were done by a single observer (PS), and the outliers were identified using the 1.5×IQR criterion for each parameter within the specimens of skull. In some specimens of skull, certain parameters exhibited outliers; these values were temporarily excluded, and the measurements were reassessed. Even after removing initial outliers, other parameters in the specimen classified as a specific skull type occasionally met the outlier criterion. Hence the authors decided to retain them in the analysis due to the limited sample size and their potential representation of natural anatomical variation. The gross appearance and structure of the LPP and its relation to the FO were observed and captured by photographs using a Nikon Z7 II full-frame mirrorless camera.

### Statistical analysis

The data was analysed, and graphs were produced using IBM SPSS Statistics (version 30). The assumption of homogeneity of variances was assessed using Levene’s test, which indicated no significant differences in variance across specimens for all parameters (*p* > 0.05). Normality of the residuals was evaluated using the Shapiro–Wilk test and visual inspection of Q–Q plots. The residuals did not show significant deviations from normality (Shapiro–Wilk *p* > 0.05), supporting the assumption of normality. Additionally, because of the moderate sample size in each group, the ANOVA is robust to minor deviations from normality. For justification, non-parametric Kruskal–Wallis tests were also conducted and yielded results consistent with the ANOVA findings (Table S1).

### Morphometric analysis of the parameters based on the type of specimens of skull

The descriptive analysis of all nine parameters was done to reveal the mean, range, and extreme values. Comparison of the parameters within the four different types of specimens of skull was conducted using a parametric one-way ANOVA, with equal sample sizes (*n* = 15 per group), and a significance level was set at *p* < 0.05. The post-hoc Tukey HSD tests were conducted to identify pairwise comparisons where significant differences in ANOVA were observed. Data was represented as mean ± standard deviation.

To assess the relationship between specific anatomical parameters of a particular type of the specimen of skull, the Pearson correlation coefficient test was employed. This statistical method was used to determine the strength and direction of the linear relationship between parameter 1 and parameters 7, 8 and 9. By calculating the Pearson correlation coefficients (r values), the analysis aimed to identify whether variations in parameter 1 were associated with proportional changes in parameters 7, 8 and 9 either in a positive or negative direction, and whether these correlations were statistically significant. The correlation strength was interpreted according to Cohen (1988): 0.00 to 0.19 - Very weak, 0.20 to 0.39 - Weak, 0.40 to 0.59 - Moderate, 0.60 to 0.79 - Strong, 0.80 to 1.00 - Very strong.

### Morphometric comparison based on laterality of skulls

Morphometric asymmetry between the left and right sides within each specimen of skull type was assessed using the Mann–Whitney U test. This approach allowed for the identification of any statistically significant laterality differences in the LPP dimensions. The analysis was conducted separately for each of the nine parameters across the specimens of skull classified as all four types (Type I-IV), with results presented as mean ± standard deviation for each side, along with corresponding p-values for the Mann–Whitney U test. The Exact Sig. (2-tailed) values were considered, with a significance threshold set at *p* < 0.05.

## Results

The morphometric analysis of the LPP across the four different types of the skull specimens revealed notable variability in the mean dimensions, ranges, and standard deviations (Table 3).

**Table 3:** Morphometric parameters analysed on the base of skull. Descriptive analysis of the morphometric parameters (1 to 9) related to the FO on the base of skull across the four different types of the specimens of skull.

Parameters	Type I Lateral			Type II Medial			Type III Direct			Type IV Removed		
	Mean ± SD	Maximum	Minimum	Mean ± SD	Maximum	Minimum	Mean ± SD	Maximum	Minimum	Mean ± SD	Maximum	Minimum
1	11.32 ± 1.70	13.75	7.94	11.87 ± 2.12	15.18	7.45	10.39 ± 3.12	16.51	5.86	10.18 ± 2.47	6.05	14.80
2	10.20 ± 2.06	14.34	6.48	12.02 ± 1.85	15.01	9.63	9.92 ± 2.31	13.49	5.75	10.16 ± 2.91	15.21	5.96
3	10.68 ± 4.06	19.14	4.92	13.14 ± 2.45	17.91	9.35	11.55 ± 2.82	16.35	7.22	10.20 ± 2.30	14.44	6.83
4	11.82 ± 2.23	16.95	9.44	12.89 ± 2.13	17.41	7.97	12.50 ± 2.60	17.09	9.44	10.46 ± 2.59	15.67	7.44
5	13.55 ± 2.36	18.68	9.39	14.21 ± 2.24	18.13	8.81	13.63 ± 2.41	17.14	8.39	11.29 ± 2.31	15.91	8.33
6	21.79 ± 2.69	29.10	18.35	22.66 ± 2.86	27.35	16.20	21.27 ± 3.07	26.12	15.60	19.83 ± 2.18	23.75	16.10
7	14.06 ± 1.68	17.53	11.43	14.31 ± 2.47	18.84	8.62	12.69 ± 2.56	17.89	9.46	13.18 ± 2.43	16.90	8.22
8	34.43 ± 2.62	39.66	30.41	34.51 ± 2.74	39.97	30.02	34.87 ± 2.95	39.40	29.82	33.36 ± 2.44	37.86	29.06
9	56.15 ± 7.52	66.60	44.70	54.40 ± 12.34	77.30	34.40	56.71 ± 9.16	75.90	44.80	54.93 ± 9.44	77.90	43.50

### Descriptive analysis of the morphometric parameters (1 to 9) across the specimens of skull classified as four types

#### Breadth of the LPP at its most distal region (1)

**Fig. 5:**
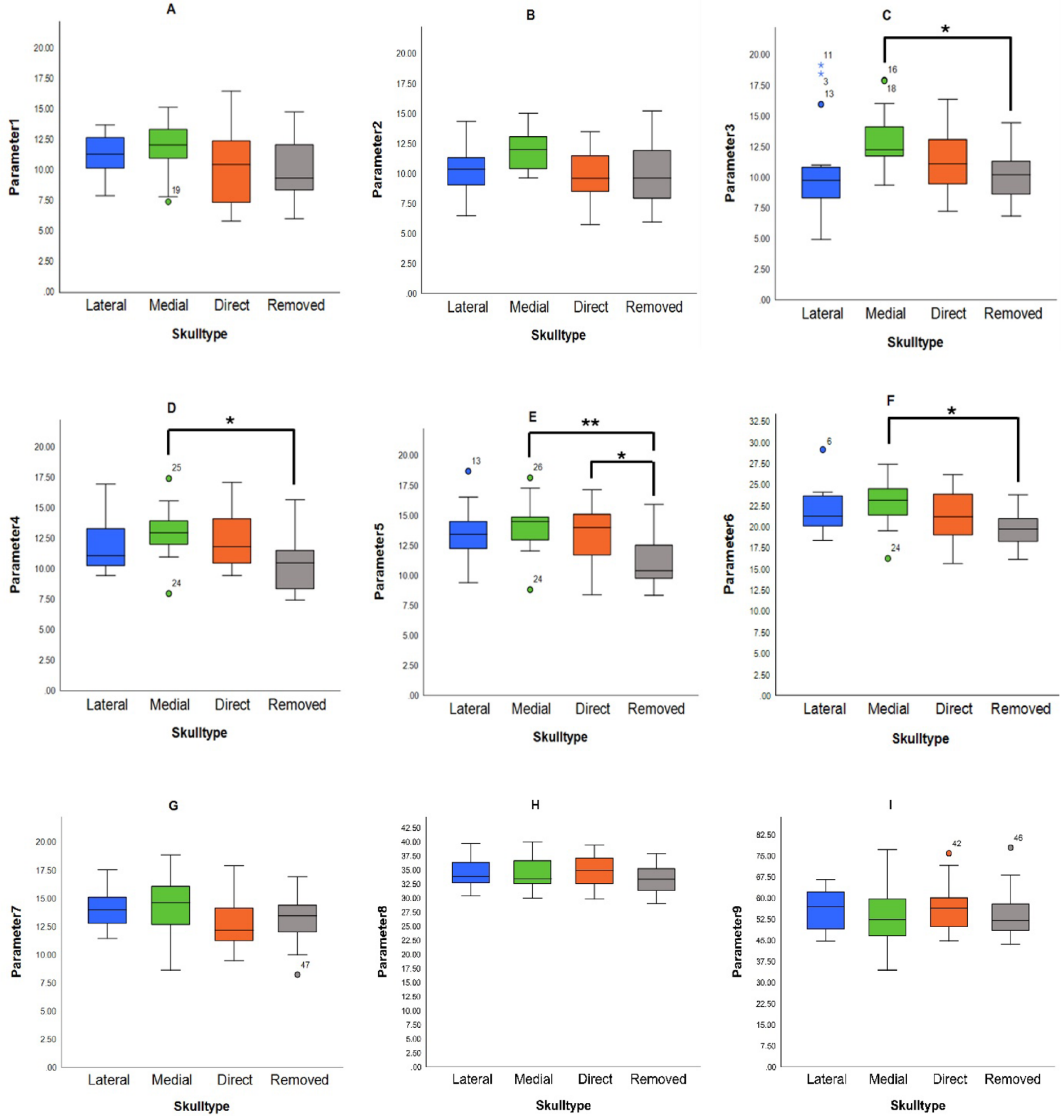
Box plot comparison of descriptive morphometric parameters of the LPP. Each panel box plots display the distribution of mean ± SD, minimum and maximum values for parameters 1–9. Outliers are marked as individual points (*n* = 15 per skull type). Asterisk *denotes significance at *p* < 0.05 and** denotes significance at *p* < 0.01.

Among the classification, the specimens of skull classified as Type II (medial) skulls exhibited the highest mean breadth (11.87 ± 2.12 mm), whereas the skull specimens classified as type IV (removed) had the lowest mean (10.19 ± 2.48 mm) (Table 1; Fig. 5A) at the most distal region of the LPP. A one-way ANOVA revealed no statistically significant differences across groups (*p* = 0.198) (Table 3).

#### Breadth at the midpoint between distal and middle regions (2)

In this parameter, the specimens of skull classified as Type II (medial) skulls demonstrated the largest mean (12.03 ± 1.85 mm), while the specimens classified as type III (direct) had the smallest mean (9.93 ± 2.31 mm). The specimens of skull classified as type IV and type I measured 10.16 ± 2.92 mm and 10.20 ± 2.06 mm, respectively (Table 1; Fig. 5B). A one-way ANOVA results did not show statistically significant differences (*p* = 0.059) (Table 3).

#### Breadth at the midpoint of the LPP (3)

Among the skull specimens, the specimens classified as type II (medial) skulls had the greatest mean (13.14 ± 2.46 mm), whereas the specimens classified as type IV (removed) had the lowest (10.20 ± 2.31 mm) mean breadth at the midpoint of the LPP (Table 3; Fig. 5C). Statistically significant intergroup differences emerged (*p* = 0.049) in one-way ANOVA (Table 4). Post hoc Tukey’s HSD analysis revealed a significant difference between the specimens of skull classified as type II and type IV (*p* = 0.046), underscoring the increased expansion of LPP at the midpoint of the specimens classified as medial type of skulls (Table S2).

#### Breadth at the midpoint between middle and proximal regions (4)

At the midpoint between the middle and proximal regions, the specimens of skull classified as type II (medial) type of skulls had the highest mean (12.90 ± 2.14 mm), with the specimens classified as type IV (removed) type showing the lowest (10.47 ± 2.60 mm) (Table 3; Fig. 5D). ANOVA revealed a statistically significant difference (*p* = 0.040) (Table 4), and Tukey’s HSD confirmed a significant pairwise difference between type II and type IV (*p* = 0.037) (Table S2).

#### Breadth at the most proximal region (5)

At the most proximal point (Table 3; Fig. 5E), the specimens of skull classified as type II (medial) maintained the highest mean breadth (14.21 ± 2.24 mm), followed by the specimens classified as type III (direct) (13.63 ± 2.42 mm) and the specimens classified as type I (lateral) (13.55 ± 2.36 mm). The specimens classified as type IV (removed) had the lowest mean (11.30 ± 2.32 mm). Statistically significant differences were detected by ANOVA (*p* = 0.006) (Table 4), with Tukey’s HSD identifying significant differences between the specimens of skull classified as type II vs. type IV (*p* = 0.006) and type III vs. type IV (*p* = 0.040) (Table S2), showing that the specimens classified as removed type had the lowest breadth at the most proximal region and the medial type are the broadest.

#### Vertical height of the LPP (6)

Among the different types of the specimens of skull, the specimens classified as Type II (medial) recorded the highest mean height of the LPP (22.67 ± 2.87 mm), while the specimens classified as type IV (removed) had the lowest height (19.83 ± 2.18 mm). The tallest height (29.10 mm) was observed in a specimen of skull classified as type I (lateral) (Table 3; Fig. 5F). ANOVA revealed a statistically significant difference (*p* = 0.045) (Table 4), with Tukey’s HSD confirming significance between the specimens classified as type II and type IV (*p* = 0.030) (Table S2) showed that the specimens classified as type II (medial) had taller mean LPPs.

#### Distance between the LPP and MPP (7)

The distance between the LPP and MPP when measured showed that the specimens of skull classified as type II (medial) demonstrated the highest mean (14.31 ± 2.47 mm), followed by the specimens of skull classified as type I (lateral) (14.06 ± 1.69 mm). The specimens of skull classified as type III and IV showed lower means (12.69 ± 2.56 mm and 13.18 ± 2.43 mm, respectively) (Table 3; Fig. 5G). Despite these differences, ANOVA did not indicate statistical significance (*p* = 0.201) (Table 4).

#### Distance from the LPP to the articular tubercle (8)

Among the different types of the specimens of skull in this study, the mean values were not varying much in this parameter. The mean values were similar across groups: the specimens of skull classified as type III (direct) − 34.87 ± 2.95 mm, the specimens of skull classified as type II (medial) − 34.51 ± 2.75 mm, the specimens of skull classified as type I (lateral) − 34.44 ± 2.62 mm, and the specimens of skull classified as type IV (removed) -33.37 ± 2.44 mm (Table 3; Fig. 5H). No statistically significant differences were detected in one-way ANOVA (*p* = 0.464) (Table 4).

#### Degree of lateral Deflection (9)

The highest mean angle of lateral deflection of the LPP was observed in the specimens of skull classified as type III (direct) (56.71° ± 9.16), with the lowest in the specimens of skull classified as type II (medial) (54.41° ± 12.35). The skull specimens classified as type IV (removed) showed the broadest range (43.50°–77.90°) (Table 3; Fig. 5I). One-way ANOVA analysis found no statistically significant differences in angular deflection between groups (*p* = 0.911) (Table 4).

**Table 4:** Morphometric and pairwise comparison between the groups. The table presents the between-group sum of squares (SS), degrees of freedom (df), mean square (MS), F-statistic (F), partial n^2^, 95% confidence intervals, cohen’s d and corresponding p-value for each parameter. Statistically significant results (*p* < 0.05) are marked with an asterisk (*). A detailed pairwise comparison table has been attached as a supplementary file table S2.

Parameters	SS (between groups)	df	MS	F	Partial *n*^2^	95% CI		*p*-value	Pairwise comparison of types	Cohen’s d	*p*-value
						Lower bound	Upper bound				
1	28.090	3	9.363	1.605	0.079	0.000	0.198	0.198	NS	2.473	NS
2	42.617	3	14.206	2.635	0.124	0.000	0.257	0.059	NS	2.437	NS
3	75.005	3	25.002	2.784	0.130	0.000	0.264	**0.049** ^*^	Medial vs. Removed	3.152	0.046*
4	51.165	3	17.055	2.956	0.137	0.000	0.272	**0.040** ^*^	Medial vs. Removed	2.539	0.037*
5	74.322	3	24.774	4.538	0.196	0.020	0.338	**0.006** ^*^	Medial vs. Removed	2.538	0.006*
									Direct vs. Removed		0.040*
6	63.500	3	21.167	2.851	0.132	0.000	0.267	**0.045** ^*^	Medial vs. Removed	2.847	0.030*
7	25.687	3	8.562	1.596	0.079	0.000	0.141	0.201	NS	2.711	NS
8	18.928	3	6.309	0.867	0.044	0.000	0.197	0.464	NS	2.369	NS
9	51.073	3	17.024	0.178	0.009	0.000	0.045	0.911	NS	9.485	NS

### Morphometric comparison between the parameters

The Pearson correlation analysis evaluated the relationships between the parameter 1 and the parameters 7, 8, 9 across the specimens of all four different types of skulls. This was to correlate whether any deviations in the distal breath of the LPP correlates with the distance within the infratemporal fossa. A statistically significant moderate positive correlation (Table 5) was found between the distal breadth of the LPP and the distance between the LPP and the medial pterygoid plate in the specimens of direct and removed types of skulls. In contrast, a statistically significant negative correlation was observed between the distal breadth of the LPP and the distance from the LPP to the anterior tubercle in the specimens of removed and lateral types of skulls. The specimens of skulls classified as lateral type showed a strong negative relationship (r -0.741, *p* = 0.002), while the specimens classified as removed type demonstrated a moderate negative correlation (Table 5). No statistically significant correlation was identified between the distal breadth of the LPP and the degree of lateral deflection in any of the types.

**Table 5:** Pearson correlation analysis between the parameters 1 and 7, 8, 9 across the four different types of the specimens of skull. The table reports correlation coefficients (*r*), 95% confidence interval, corresponding *p*-values, and qualitative interpretations of correlation strength. Asterisks denote statistical significance (*p* < 0.05 *; *p* < 0.01 **).

Comparison between the parameters	Correlation strength (*r*)	95% CI		*p* value	Strength of correlation
		Lower bound	Upper bound		
Correlation of the parameters 1 and 7					
**Direct**	0.549*	0.051	0.828	0.034*	Moderately positive
**Medial**	0.498	-0.019	0.805	0.059	Moderately positive
**Removed**	0.596*	0.121	0.849	0.019*	Moderately positive
**Lateral**	0.133	-0.407	0.604	0.635	Very weak positive
**Correlation of the parameters 1 and 8**					
**Direct**	-0.279	-0.692	0.273	0.315	Weak negative
**Medial**	-0.377	-0.745	0.167	0.166	Weak negative
**Removed**	-0.584*	-0.844	-0.102	0.022*	Moderately negative
**Lateral**	-0.741**	-0.908	-0.369	0.002**	Strongly negative
**Correlation of the parameters 1 and 9**					
**Direct**	-0.015	-0.523	-0.523	0.958	Very weak negative
**Medial**	0.025	-0.493	0.531	0.928	Very weak positive
**Removed**	-0.185	-0.637	0.361	0.509	Very weak negative
**Lateral**	0.139	-0.402	0.608	0.621	Very weak positive

### Morphometric comparison based on laterality

The statistical significance between the right and the left sides of skulls was analysed by Mann-Whitney’s test. Across all nine parameters, no statistically significant differences were observed between the left and right sides for any of the parameters in the four different four different types, with p value exceeding 0.05 (Table S3, S4, S5, S6).

### Sensitivity analysis of the results

Using the 1.5×IQR box-plot rule, outliers ranged from 0% to 33.3% across groups (Table S7). After excluding all cases flagged as outliers, the overall pattern of results remained largely consistent. In ANOVA, the specimens of skull classified as removed type continued to have the lowest values across LPP-three, LPP-four, and LPP-five, while the specimens of skull classified as medial group had higher values. Differences that were statistically significant became marginally non-significant (p-values ranging from 0.059 to 0.089) for LPP-three, LPP-four, and LPP-five, though the specimens of skull classified as medial type remains robust. For the height of the LPP, the effect of skull type was sensitive to outliers and became non-significant (*p* = 0.176) after outlier removal. The Pearson correlation, which was central to this study, demonstrated robust results, maintaining consistency even after outliers were excluded. Overall, the sensitivity analyses excluding these outliers indicated that the main findings remained largely consistent, suggesting that the results are generally robust to extreme values. Outliers were retained in the main analysis because they represent plausible observations, and this study was also limited by the availability of the specimens of skulls classified as medial and removed types.

## Discussion

This study, building upon the anatomical classifications established by Iwanaga et al. (2022)^4^, provides a comprehensive morphometric analysis of the LPP in all four different specimens of skull, with specific focus on its implications for percutaneous access to the FO through the ITF. Prior studies emphasised the high interindividual variability within the pterygoid region^12^. While valuable, those studies did not differentiate by skull type, which may obscure patterns of morphometric predisposition. Although the posterior border of the LPP is an important anatomical landmark, classifying specimens of skull based solely on the positional relationship to the FO as proposed in previous studies^6,9,13^ does not primarily helpful for assessing access to the FO. Additionally, these studies offer limited guidance for evaluating the ITF corridor, which plays a more critical role in clinical decision making during percutaneous procedures. As the ITF surgical corridor is bordered medially by the LPP and laterally by the zygomatic arch, any deviations or hypertrophy in LPP can influence procedural success (Fig. 2). Crucially, this present study contributes a five-point morphometric analysis of the LPP, capturing dimensional changes along its full length. Kapur (2003) have similarly noted that the bulk and orientation of the LPP play a critical role in guiding mandibular nerve block techniques^11^.

Though there are studies on LPP, they are mostly reconstructed the CT images of the patients. Kwon et al. made the measurements of the breadth of the LPP in three regions: in the superior region were 17.62 ± 2.4 mm on the right side and 17.70 ± 2.49 mm on the left side. The middle portion showed 13.38 ± 3.38 mm on the right and 13.04 ± 3.39 mm on the left. The inferior segment measured 13.38 ± 4.03 mm on the right and 14.06 ± 4.19 mm on the left^14^. In the present study, the greater LPP proximal breadth was 14.21 ± 2.24 mm, at the midpoint was 13.14 ± 2.45 mm and on the distal region was 11.87 ± 2.12 mm. Pterygoid height made at the anterior border of the LPP was 28.50 ± 2.41 mm on the right and 28.76 ± 2.67 mm on the left according to the CBCT images^14^. In the present study, the highest mean height of the posterior border of the LPP was 22.66 ± 2.86 mm. These differences may be attributed to different factors. Kwon et al. obtained their data from 3D-reconstructed CT images of patients, and their measurements were made on CBCT models established within a three-dimensional coordinate system, rather than directly on the bony margins as in the present study. In their analysis, the landmarks were primarily oriented toward the posterior wall of the maxilla, which differs from the reference points used in this study. Furthermore, population characteristics may have influenced the results; the study sample of Kwon et al. comprised Korean adults, whereas the present study examined specimens of skull from a different demographic background. Because craniofacial dimensions vary with ethnicity, sex, and age, such variation could also contribute to the observed differences between the two datasets^14^.

To our knowledge, this is the first study to quantify five sectional breadth measurements of the LPP in dry human skulls, highlighting its spatial relationship with adjacent landmarks like the articular tubercle of the zygomatic arch and the medial pterygoid plate mainly focussing on the entry to the ITF. The findings of this study demonstrate that the specimens of skull classified as medial type exhibit consistently statistically significant differences in both the height and the breadth of the LPP, particularly from the distal end to the midpoint, when compared to other skull types. Additionally, the specimens of skull classified as medial type showed a greater breadth in the rest of the two sections of LPP though not statistically significant. Despite this uniform hypertrophy or enlargement of the LPP in the specimens of skull classified as medial type, no statistically significant reduction was observed in the space between the LPP and the AT which denotes the entry to the ITF, indicating that increased LPP bulk in the specimens of skull classified as medial type does not necessarily compromise the surgical corridor required for percutaneous approaches to the FO. This also suggests that the increased breadth in these skulls is likely oriented more vertically and the medial position of the LPP does not translate into increased lateral displacement that would impact the AT-LPP spatial relationship. Notably, the anatomical configuration of the LPP in the specimens of skull classified as medial type may offer a potential clinical advantage. The more robust and medially oriented position of the LPP may facilitate more direct needle guidance toward the FO, potentially minimizing the risk of inadvertent puncture of adjacent foramina that house critical neurovascular structures.

In contrast to these observations, the present study highlights that the specimens of skull classified as lateral type showed a statistically significant stronger negative correlations between distal LPP breadth and its distance to the articular tubercle (p value = − 0.002). This suggests that an increase in distal breadth of the LPP in the specimens of skull classified as lateral type is associated with a decrease in distance from LPP and the AT. This lateral displacement effectively narrows the surgical corridor of the ITF, complicating the needle’s trajectory and increasing the risk of obstruction or injury to adjacent structures. These specimens represent what could be termed the “obstructive type” in terms of both access and safety. These specimens of skull classified as lateral type, despite lacking a broader or higher LPP, have the plate positioned more laterally in relation to the FO, resulting in a narrower ITF space.

The specimens of skull classified as removed type presents the most challenging configuration, as they lack a distinct bony guidance landmark due to atypical LPP morphology, specifically, a deficient posterior border of the LPP. As noted by Iwanaga et al. (2022), these skulls carry the highest risk for procedural error and, according to the present study (*p* = 0.022), it also provide the least predictable access corridor, supporting their clinical classification as obstructive types^4^. Furthermore, an increase in the distal breadth of the LPP demonstrated a statistically significant, moderately positive correlation with the distance between the LPP and the MPP (*p* = 0.019), indicating that as the distal breadth increases, the LPP–MPP distance also increases, whereas the distance between the LPP and the articular tubercle decreases. The specimens of skull classified as lateral and removed types representing approximately 50% of the population according to Iwanaga et al. (2022)^4^ and 53.7% as reported by Triantafyllou et al. (2025)^15^ were both found to exhibit a narrowed ITF space, which may further complicate percutaneous access to the FO.

This study shows that the LPP exhibits significant morphological variations across the four different classifications of the specimens of skull, with detailed projections occurring along its entire length. These differences can directly impact the safety and effectiveness of infratemporal fossa cannulation. The specimens of skull classified as medial type, despite having broader and higher LPPs, maintain a favourable surgical corridor due to limited lateral deflection. In contrast, the specimens of skull classified as lateral and removed types may pose greater procedural challenges due to obstructive LPP positioning. This anatomically suggests that surgical access in such configurations of skulls could be more limited, although this interpretation remains hypothetical. Confirmation requires measurement of the same morphological types in the clinical setup using CBCT series and correlation with intraoperative access outcomes before firm clinical recommendations can be made.

### Limitations

Several limitations of this study should be acknowledged. The use of dry human skulls excluded the assessment of soft tissue structures such as the lateral pterygoid muscle and surrounding vascular anatomy, limiting direct clinical translation. In this study, percutaneous access to the FO is influenced by both bony and soft tissue factors; thus, skeletal morphology alone cannot fully predict procedural difficulty. Linear calliper-based assessments are inherently two-dimensional and may not capture spatial curvatures relevant to needle trajectory. The lack of imaging modalities such as CBCT prevented three-dimensional modelling of the infratemporal corridor. Another major limitation concerns the absence of demographic information for the specimens as well as random selection of skulls. The unknown sex, age, and ethnicity of skulls precluded assessment of population-based variation, thereby limiting generalizability to clinical or demographic subgroups. Moreover, potential collection bias inherent in institutional bone sets where specimens may not represent population diversity could influence the morphometric trends observed. The same methodological approach used in this study could yield more clinically transferable data if applied to skull collections with defined demographic information (sex, age, and ethnicity) or in combination with imaging based three-dimensional models. Additionally, outliers were retained in the dataset to preserve the natural variability of anatomical morphology. It may have increased dispersion estimates and slightly widened confidence intervals. The comparison of outcomes with and without outlier exclusion was done to assess the influence of it on overall variables in the present study. Nevertheless, excluding outliers in future studies particularly when larger sample sizes are available could help refine statistical precision and minimize potential distortion of mean values.

## Supplementary Information

Below is the link to the electronic supplementary material.

Supplementary Material 1

## Data Availability

The relevant data are included within the article as well as in the form of a supplementary file. Additional data supporting the findings including the complete raw measurement dataset and SPSS analysis scripts used in this study have been deposited in Figshare (DOI: 10.6084/m9.figshare.30279310).
