# Supplementary material for: Anatomical variability of the lateral pterygoid plate and its influence on the dimensions of infratemporal fossa in human skulls
Source: Sci Rep. 2025 Nov 28;15:42606. doi: 10.1038/s41598-025-26776-6 (PMC12663222; doi:10.1038/s41598-025-26776-6)
Supplement: Supplementary file 1 — Supplementary Material 1 [file 41598_2025_26776_MOESM1_ESM.docx]

**Supplementary file**

**Title: Anatomical variability of the lateral pterygoid plate and its influence on the dimensions of infratemporal fossa in human skulls**

Pavan Sohal 1, Aisha Zeeshan 2, Femina Sam 2,3*

1 Department of Biomedical Sciences, College of Medicine and Health, University of Birmingham, United Kingdom, B15 3TT

2 Human Anatomy Unit, Department of Biomedical Sciences, School of Infection, Inflammation and Immunology, College of Medicine and Health, University of Birmingham, United Kingdom, B15 3TT

3 Department of Anatomy, Christian Medical College, Vellore, Affiliated to the Tamil Nadu Dr. M.G.R. Medical University, Chennai, India, 632 002

**Corresponding author***

Femina Sam

Demonstrator,

Human Anatomy Unit,

Department of Biomedical Sciences,

School of Infection, Inflammation and Immunology,

College of Medicine and Health,

University of Birmingham,

United Kingdom,

B15 3TT

Email id: [f.s.janetsammohanroypauljosephinesudha@bham.ac.uk](mailto:f.s.janetsammohanroypauljosephinesudha@bham.ac.uk)

| Parameters | Levene statistic (sig.) | Shapiro- Wilk (Sig.) Test | Kruskal- Wallis Test |
| --- | --- | --- | --- |
| 1 | 0.164 | 0.280 | 0.180 |
| 2 | 0.310 | 0.448 | **0.046*** |
| 3 | 0.406 | 0.067 | **0.008*** |
| 4 | 0.547 | 0.149 | **0.026*** |
| 5 | 0.565 | 0.390 | **0.012*** |
| 6 | 0.924 | 0.538 | **0.030*** |
| 7 | 0.665 | 0.287 | 0.148 |
| 8 | 0.817 | 0.848 | 0.540 |
| 9 | 0.819 | 0.313 | 0.857 |

**Table S1. Assumption checks of the parameters across all four types of skulls and non-parametric validation for ANOVA.** This table provides the p-values from Levene’s test (homogeneity of variances), Shapiro–Wilk test (normality of residuals), and Kruskal–Wallis test (non-parametric ANOVA alternative). Asterisks (*****) indicate statistically significant differences (*p* < 0.05)

| Comparison between the groups | Difference in means | p-value | 95% Confidence Intervals | |
| --- | --- | --- | --- | --- |
|  |  |  | **Lower** | **Upper** |
| Breadth at the midpoint of the LPP (Parameter 3) |  |  |  |  |
| Medial Vs Lateral | 2.45733 | 0.124 | -4.401 | 5.3548 |
| Direct Vs Lateral | 0.86933 | 0.857 | -2.0281 | 3.7668 |
| Lateral Vs Removed | 0.48067 | 0.971 | -2.4168 | 3.3781 |
| Medial Vs Direct | 1.58800 | 0.473 | -1.3095 | 4.4855 |
| Medial Vs Removed | 2.93800* | **0.046*** | 0.0405 | 5.8355 |
| Direct Vs Removed | 1.35000 | 0.608 | -1.5475 | 4.2475 |
| Breadth of the LPP at the midpoint between the middle and proximal regions (Parameter 4) |  |  |  |  |
| Medial Vs Lateral | 1.07133 | 0.616 | -3.3938 | 1.2512 |
| Direct Vs Lateral | 0.67600 | 0.867 | -1.6465 | 2.9985 |
| Lateral Vs Removed | 1.35800 | 0.416 | -0.9645 | 3.6805 |
| Medial Vs Direct | 0.39533 | 0.969 | -1.9272 | 2.7178 |
| Medial Vs Removed | 2.42933* | **0.037*** | 0.1068 | 4.7518 |
| Direct Vs Removed | 2.03400 | 0.106 | -0.2885 | 4.3565 |
| Breadth of the LPP at the proximal-most region of the LPP (Parameter 5) |  |  |  |  |
| Medial v Lateral | 0.65733 | 0.867 | -1.6017 | 2.9164 |
| Direct v Lateral | 0.7933 | 1.000 | -2.1797 | 2.3384 |
| Lateral v Removed | 2.25707 | 0.050 | -0.0020 | 4.5161 |
| Medial v Direct | 0.57800 | 0.905 | -1.6811 | 2.8371 |
| Medial v Removed | 2.91440* | **0.006*** | 0.6553 | 5.1735 |
| Direct v Removed | 2.33640* | **0.040*** | 0.0773 | 4.5955 |
| Height of the LPP (Parameter 6) |  |  |  |  |
| Medial v Lateral | 0.87000 | 0.818 | -1.7646 | 3.5046 |
| Direct v Lateral | -0.52000 | 0.953 | -3.1546 | 2.1146 |
| Lateral v Removed | 1.96400 | 0.210 | -0.6706 | 4.5986 |
| Medial v Direct | 1.39000 | 0.506 | -1.2446 | 4.0246 |
| Medial v Removed | 2.83400* | **0.030*** | 0.1994 | 5.4686 |
| Direct v Removed | 1.44400 | 0.473 | -1.1906 | 4.0786 |

**Table S2. Pairwise comparisons of mean linear measurements of the LPP across four skull types (Medial, Direct, Lateral, and Removed).**This table summarizes the difference in means, corresponding *p*-values, and 95% confidence intervals for the following parameters 3. Breadth at the midpoint of the LPP, 4. Breadth between the midpoint and proximal regions, 5. Breadth at the proximal-most region and 6. Height of the LPP. Asterisks (*****) indicate statistically significant differences (*p* < 0.05).

| U statistic | Exact P value | Parameter | Mean ±SD | | Minimum | | Maximum | |
| --- | --- | --- | --- | --- | --- | --- | --- | --- |
|  |  |  | **Left** | **Right** | **Left** | **Right** | **Left** | **Right** |
| 24.000 | 0.776 | 1 | 11.35 ±1.72 | 11.27 ±1.84 | 7.94 | 9.18 | 13.75 | 13.34 |
| 23.000 | 0.689 | 2 | 9.94 ± 2.39 | 10.60± 1.56 | 6.48 | 8.76 | 14.34 | 12.57 |
| 24.000 | 0.776 | 3 | 10.08 ±3.84 | 11.58 ±4.58 | 4.92 | 6.84 | 19.14 | 18.43 |
| 21.000 | 0.529 | 4 | 11.30 ±2.88 | 12.23 ±1.99 | 9.83 | 9.44 | 16.95 | 14.93 |
| 20.000 | 0.456 | 5 | 13.03 ± .89 | 14.34± 2.94 | 9.39 | 10.37 | 15.81 | 18.68 |
| 17.000 | 0.272 | 6 | 22.45 ±3.11 | 20.81± 1.67 | 18.35 | 19.32 | 29.10 | 23.62 |
| 22.000 | 0.607 | 7 | 13.82 ±1.59 | 14.41 ±1.90 | 11.43 | 12.28 | 16.47 | 17.53 |
| 22.000 | 0.607 | 8 | 34.75 ±3.11 | 33.95±1.81 | 30.41 | 32.07 | 39.66 | 36.97 |
| 15.000 | 0.181 | 9 | 53.66 ±8.19 | 59.88± 4.81 | 44.70 | 53.30 | 66.60 | 66.00 |

**Table S3. Descriptive and statistical analysis of the parameters for Type I – lateral.** Displaying the distribution of mean ± SD, minimum and maximum values for parameter 1-8 (mm) and 9 (°) as left to right side comparisons and the U-statistic and p-value for each corresponding parameter. Sample size is shown for each side; no statistically significant difference between left and right sides for parameters tested (p < 0.05)

| U statistic | Exact P value | Parameter | Mean ±SD | | Minimum | | Maximum | |
| --- | --- | --- | --- | --- | --- | --- | --- | --- |
|  |  |  | **Left** | **Right** | **Left** | **Right** | **Left** | **Right** |
| 20.000 | 0.397 | 1 | 11.67± 2.21 | 12.09 ±2.15 | 7.45 | 7.85 | 15.18 | 14.10 |
| 13.000 | 0.094 | 2 | 11.28± 1.83 | 12.88 ±1.59 | 9.63 | 10.26 | 15.01 | 14.92 |
| 20.000 | 0.397 | 3 | 12.24± 1.57 | 14.16 ±2.98 | 9.35 | 11.05 | 14.77 | 17.91 |
| 17.000 | 0.232 | 4 | 12.46± 2.65 | 13.39 ±1.39 | 7.97 | 11.27 | 17.41 | 15.57 |
| 16.500 | 0.189 | 5 | 13.47± 2.48 | 15.05 ±1.73 | 8.81 | 12.86 | 17.27 | 18.13 |
| 25.000 | 0.779 | 6 | 22.22 ±3.13 | 22.17 ±2.67 | 16.20 | 19.47 | 25.64 | 27.35 |
| 26.000 | 0.867 | 7 | 14.00 ±2.59 | 14.65 ±2.48 | 8.62 | 12.13 | 16.59 | 18.84 |
| 25.000 | 0.779 | 8 | 34.32 ±3.32 | 34.72 ±2.15 | 30.02 | 32.37 | 39.97 | 37.54 |
| 19.000 | 0.336 | 9 | 51.76 ±13.07 | 27.42 ± 1.67 | 34.40 | 39.70 | 77.30 | 72.50 |

**Table S4. Descriptive and statistical analysis of the parameters for Type II - medial.** Displaying the distribution of mean ± SD, minimum and maximum values for parameter 1-8 (mm) and 9 (°) as left to right side comparisons and the U-statistic and p-value for each corresponding parameter. Sample size is shown for each side, no statistically significant difference between left and right sides for parameters tested (p < 0.05)

| U statistic | Exact P value | Parameter | Mean ±SD | | Minimum | | Maximum | |
| --- | --- | --- | --- | --- | --- | --- | --- | --- |
|  |  |  | **Left** | **Right** | **Left** | **Right** | **Left** | **Right** |
| 24.000 | 0.694 | 1 | 10.33±3.27 | 10.44±3.21 | 6.52 | 5.86 | 15.05 | 16.51 |
| 22.000 | 0.536 | 2 | 10.42±2.48 | 9.49 ± 2.22 | 6.79 | 5.75 | 13.49 | 13.41 |
| 19.000 | 0.336 | 3 | 12.39±2.98 | 10.81±2.64 | 8.76 | 7.22 | 16.35 | 16.03 |
| 27.000 | 0.955 | 4 | 12.7 ± 3.19 | 12.29±2.16 | 9.44 | 9.61 | 17.09 | 16.30 |
| 26.500 | 0.867 | 5 | 13.66±3.00 | 13.60±1.98 | 8.39 | 11.04 | 17.14 | 16.57 |
| 26.000 | 0.867 | 6 | 21.52±2.61 | 21.05±3.59 | 18.98 | 15.60 | 25.27 | 26.12 |
| 27.000 | 0.955 | 7 | 12.66±2.65 | 12.71±2.66 | 9.46 | 9.50 | 17.89 | 17.16 |
| 27.000 | 0.955 | 8 | 34.85±2.99 | 34.89±3.12 | 31.54 | 29.82 | 39.40 | 38.59 |
| 27.000 | 0.955 | 9 | 56.97±12.54 | 56.48±5.72 | 44.90 | 44.80 | 75.90 | 63.20 |

**Table S5. Descriptive and statistical analysis of the parameters for Type III – direct.** Displaying the distribution of mean ± SD, minimum and maximum values for parameter 1-8 (mm) and 9 (°) as left to right side comparisons and the U-statistic and p-value for each corresponding parameter. Sample size is shown for each side, no statistically significant difference between left and right sides for parameters tested (p < 0.05)

| U statistic | Exact P value | Parameter | Mean ±SD | | Minimum | | Maximum | |
| --- | --- | --- | --- | --- | --- | --- | --- | --- |
|  |  |  | **Left** | **Right** | **Left** | **Right** | **Left** | **Right** |
| 24.000 | 0.694 | 1 | 10.30± 2.34 | 10.08± 2.74 | 7.02 | 6.05 | 12.37 | 14.80 |
| 22.000 | 0.536 | 2 | 10.54± 3.28 | 9.82± 2.74 | 5.96 | 6.52 | 15.15 | 15.21 |
| 19.000 | 0.336 | 3 | 10.42± 2.72 | 10.01± 2.05 | 7.27 | 6.83 | 14.44 | 13.81 |
| 27.000 | 0.955 | 4 | 10.72± 3.03 | 10.24± 2.34 | 7.44 | 7.49 | 15.67 | 14.96 |
| 26.500 | 0.867 | 5 | 11.12± 2.70 | 11.45± 2.10 | 8.33 | 9.52 | 15.91 | 15.06 |
| 26.000 | 0.867 | 6 | 20.6 ± 2.37 | 19.15 ± 1.88 | 18.04 | 16.10 | 23.75 | 22.00 |
| 27.000 | 0.955 | 7 | 13.25 ±3.24 | 13.12 ± 1.67 | 8.22 | 10.47 | 16.90 | 15.67 |
| 27.000 | 0.955 | 8 | 33.41± 2.72 | 33.33 ± 2.36 | 30.885 | 29.06 | 37.86 | 36.42 |
| 27.000 | 0.955 | 9 | 53.35 ±6.41 | 56.31±11.77 | 46.90 | 43.50 | 66.10 | 77.90 |

**Table S6. Descriptive and statistical analysis of the parameters for Type IV – removed.** Displaying the distribution of mean ± SD, minimum and maximum values for parameter 1-8 (mm) and 9 (°) as left to right side comparisons and the U-statistic and p-value for each corresponding parameter. Sample size is shown for each side, no statistically significant difference between left and right sides for parameters tested (p < 0.05)

| **Parameters** | **Number of outliers** | **%** |
| --- | --- | --- |
| **1** | 1 | 6.7% |
| **2** | 0 | 0 |
| **3** | 5 | 33.3 % |
| **4** | 2 | 13.3 % |
| **5** | 3 | 20% |
| **6** | 2 | 13.3% |
| **7** | 1 | 6.7 % |
| **8** | 0 | 0 |
| **9** | 2 | 13.3 % |

**Table S7. Outliers per parameter**
Table showing the number and percentage of outliers in each group based on the 1.5×IQR box-plot rule.
